# ALK-positive gastric inflammatory myofibroblastic tumor in an adult with familial adenomatous polyposis and diffuse fundic polyposis

**DOI:** 10.1186/s13000-017-0645-0

**Published:** 2017-09-18

**Authors:** Jun Fan, Bo Huang, Xiuping Yang, Ming Yang, Jun He, Xiu Nie

**Affiliations:** Department of pathology, Union Hospital, Tongji Medical College, Huazhong University of Science and Technology, Wuhan, 430022 China

**Keywords:** Inflammatory myofibroblastic tumors, Familial adenomatous polyposis, Gastric fundic gland polyposis, Anaplastic lymphoma kinase

## Abstract

**Background:**

Inflammatory myofibroblastic tumor (IMT) of the stomach is extremely rare in adults and exhibits a variable biological behavior that ranges from frequently benign lesions to more aggressive variants. Here we report a case of gastric IMT with lymph node metastasis in an adult who had undergone total colectomy for familial adenomatous polyposis (FAP).

**Case presentation:**

A 37-year-old man presented gradual-onset epigastric discomfort; he had undergone total colectomy for FAP 6 years before. The upper endoscopy revealed diffuse polyposis in the body of stomach and a submucosal protruding tumor of approximately 4.5 × 3.5 cm in the gastric angular incisure, appearing like gastrointestinal stromal tumor. Histology after surgery verified the diagnosis of fundic gland polyposis (FGPs) and gastric IMT with lymph node metastasis. Both the primary IMT tissue and its metastatic lesion but not the FGP or FAP tissue were positive for anaplastic lymphoma kinase (ALK) on immunohistochemical staining. Fluorescent in situ hybridization confirmed the existence of ALK rearrangement in IMT tissues. However, the patient exhibited no abnormalities in microsatellite instability or mismatch repair-system components, including MSH6, MSH2, MLH1 and PMS2, in IMT, FGP or FAP tissue.

**Conclusions:**

This case allowed for exploring the relationship among IMT, FGP and FAP and indicates that gastric IMT should be considered in the diagnosis of a gastric mass in patients with FAP. ALK may be a useful biomarker in the diagnosis of IMT and its metastatic lesions.

**Electronic supplementary material:**

The online version of this article (doi:10.1186/s13000-017-0645-0) contains supplementary material, which is available to authorized users.

## Background

Inflammatory myofibroblastic tumor (IMT) is a rare mesenchymal neoplasm characterized by spindle cells with myofibroblastic differentiation admixed with inflammatory infiltrates of lymphocytes, plasma cells, and eosinophils [1]. IMT typically shows benign or intermediate clinical behavior and rarely presents metastasis [2]. IMT most frequently affects the lung and is extremely rare in the stomach of adults [3–5].

The etiology and pathogenesis of IMT remains unclear. Epstein-Barr virus (EBV) infection and immune dysregulation are reported to associate with some cases of IMT [6, 7]. At molecular level, approximately 50% of IMT cases contain rearrangements involving the anaplastic lymphoma kinase (ALK) gene, leading to constitutive activation of the tyrosine kinase, as well as positive immunohistochemical staining for ALK [8], which distinguishes IMT from gastrointestinal stromal tumor (GIST), leiomyoma, and leiomyosarcoma.

Here we report a case of ALK-positive IMT with lymph-node metastasis in the stomach of an adult with a history of familial adenomatous polyposis (FAP). This may be the first gastric IMT case with metastasis in an adult showing gastric fundic gland polyposis (FGPs) after total colectomy for FAP.

## Case presentation

**Fig. 1:**
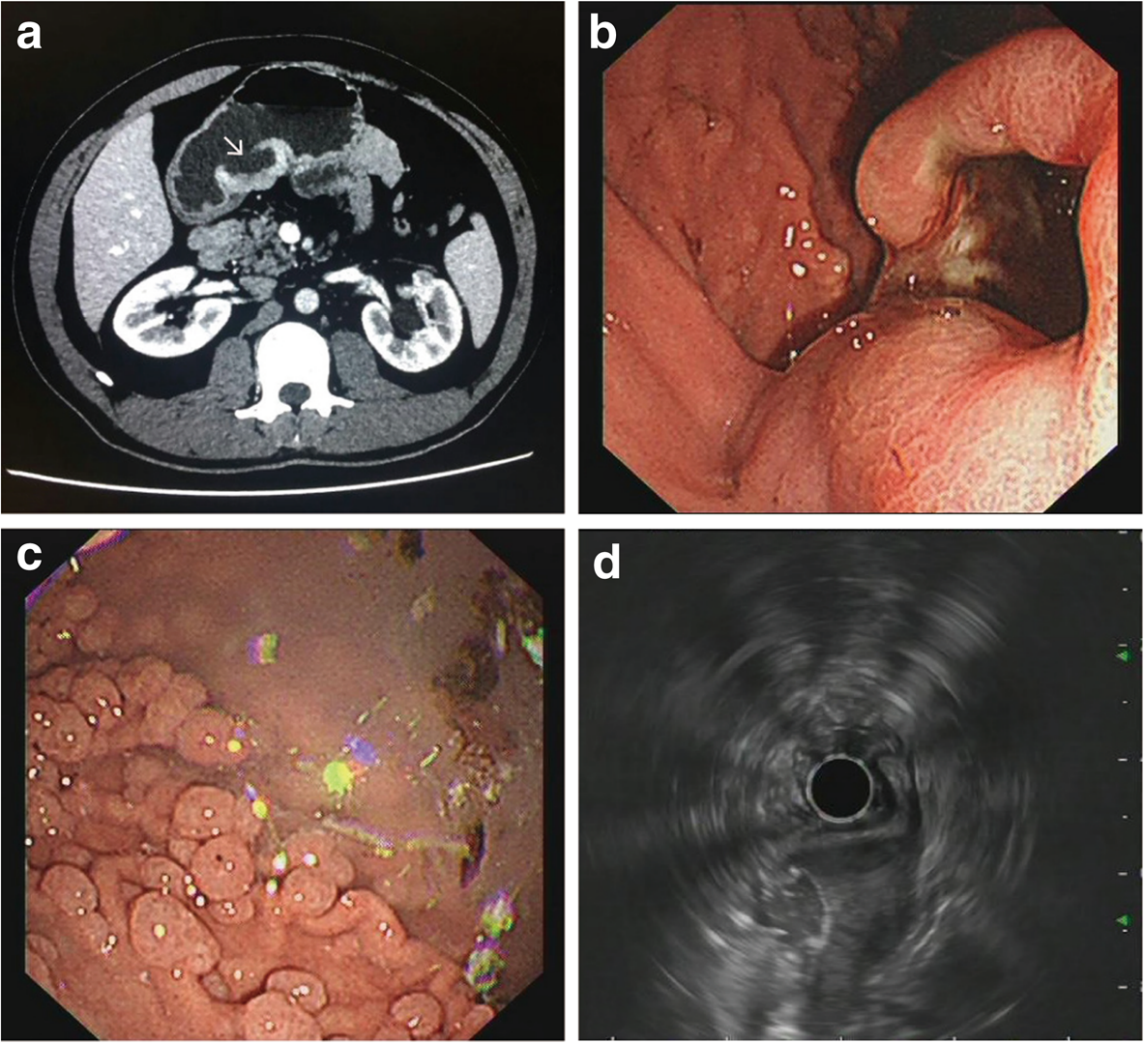
CT, endoscopy and ultrasonography examination. **a** Abdominal CT shows a broad-based, protruding mass with deep ulceration on the surface (*arrow*), arising from the sub-mucosal layer of the gastric antrum along the lesser curvature of the stomach. **b** Endoscopy shows a solid protruding mass with deep surface ulceration in the gastric angular incisures and **c** numerous polyps at the fundus and body of the stomach. **d** Electronic ultrasonic endoscopy showed the solid protruding mass

A 37-year-old man presented upper abdominal pain for 1 month; he had undergone total colectomy for FAP 6 years ago. Abdominal Computed tomography (CT) revealed a broad-based mass measuring 4.4 × 2.2 cm located in the wall of the gastric antrum along the lesser curvature and protruding to the cavity, with an ulcer on the surface (Fig. 1a). Endoscopy verified the solid protruding mass with surface ulceration (Fig. 1b) and revealed numerous polyps at the fundus and body of the stomach (Fig. 1c). Electronic ultrasonic endoscopy indicated that the lesion most likely originated from a muscular layer and GIST was under consideration for diagnosis (Fig. 1d). Laboratory evaluation revealed an increase in blood level of neuron-specific enolase (28.35 μg/L, normally <16.3 μg/L). Total gastrectomy was performed and lymph nodes around the stomach were collected.

**Fig. 2:**
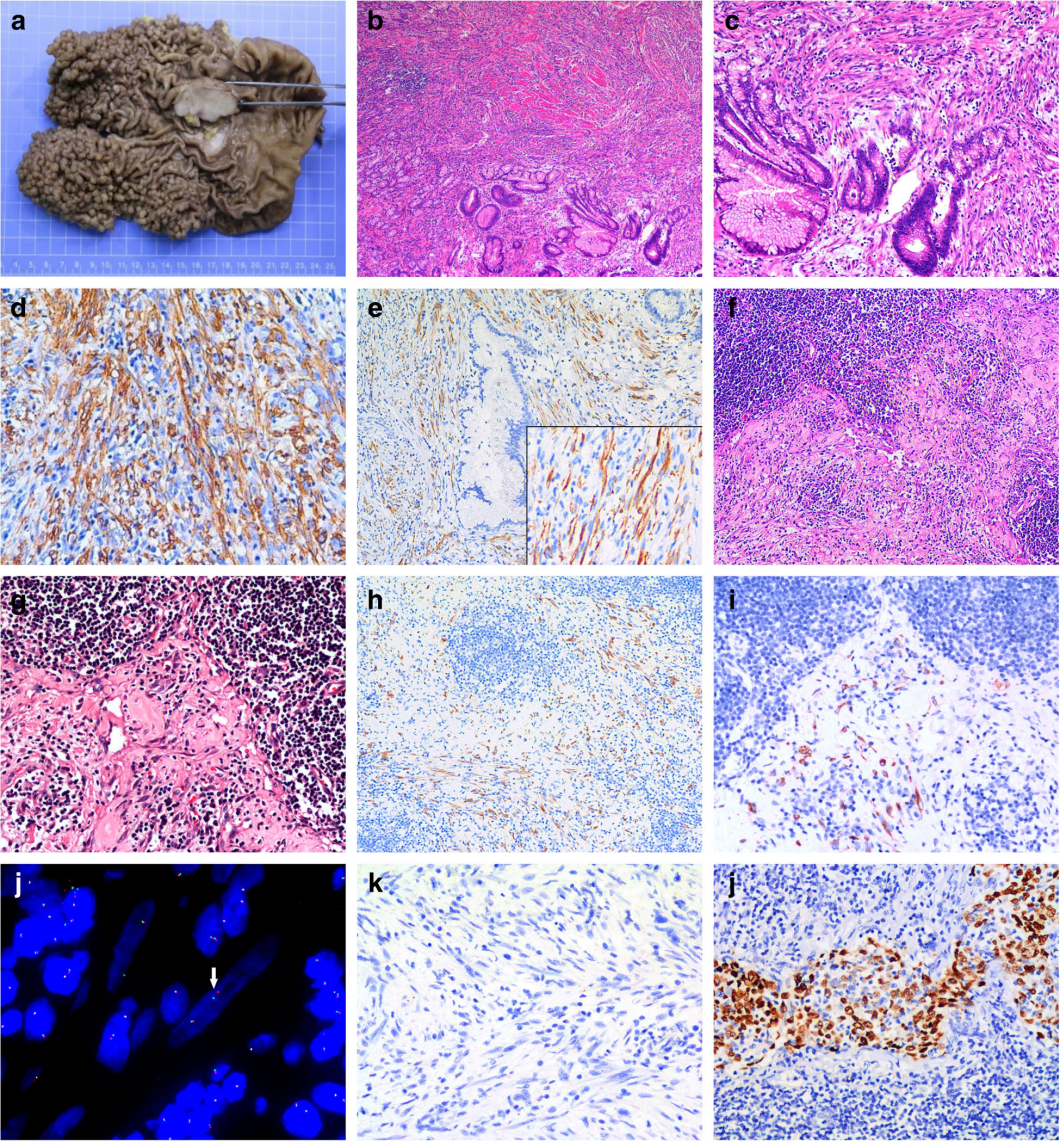
Gross and microscope observations. A protruding mass with white cut surface in the gastric antrum and many fundic gland polyps diffusely distributed in the cardia and body of stomach (**a**). Microscopic section of the mass characterized by spindle cells arranged in a fascicular pattern with prominent chronic inflammatory cell infiltrates (**b**, H&E stain, ×40). High power microscopy of spindle-shaped cells admixed with infiltrating lymphocytes and plasma cells (**c**, H&E stain, ×100). The tumor cells were positive in cytoplasm for smooth muscle actin (SMA) (**d**) and anaplastic lymphoma kinase (ALK) (**e**) (Immunohistochemical stain, ×100, right bottom ×200). Five of 19 lymph nodes adjacent to stomach contain metastatic tumor cells (**f**, H&E stain, ×100; **g**, ×200), and were positive for ALK (**h**, Immunohistochemical stain, ×100; **i**, ×200). Fluorescent in situ hybridization analysis of ALK rearrangement in inflammatory myofibroblastic tumor using Vysis break apart probe kit (**j**, 1000×) and chromogenic in situ hybridization analysis of Epstein-Barr virus-encoded small RNAs using Leica Bond ready-to-use probe in inflammatory myofibroblastic tumor (**k**, 1000×) and nasopharyngeal carcinoma as positive control (**l**, 1000×)

On gross examination, the mass was 4.5 × 3.5 cm with a white cut surface and invaded the muscular layer of the gastric wall. Hundreds of polyps with basal diameter 0.2 to 1 cm were diffusely distributed in the cardia and body of the stomach with an area of 12.5 × 11.5 cm (Fig. 2a). Histologically, myofibroblastic spindle cells are arranged in a fascicular pattern with prominent chronic inflammatory cells infiltrating the collagenous stroma (Fig. 2b). With high power examination, spindle-shaped cells had plump vesicular nuclei and eosinophilic cytoplasm and were admixed with infiltrating lymphocytes and plasma cells (Fig. 2c). The mitotic count was 1/10 high power fields. Cellular atypia was focal and seemed reactive, mostly observed in the vicinity of ulcerative superficial portions. Histology of the polyps in gastric cardia and body showed proliferation of FGPs with some cystic dilatation, and the diagnosis was FGP without dysplasia.

On immunohistochemistry, tumor cells were positive for vimentin, smooth muscle actin (Fig. 2d), ALK (Fig. 2e) and showed focal positivity for desmin and CD34 but were negative for pan-cytoceratin, CD117, DOG-1, S-100, h-caldesmon, CD21, CD23 and CD30. Ki-67 labeling index was estimated at 10% and the ratio of immunoglobulin G4 (IgG4)- to IgG-positive lymphocytes was <0.4 (Additional file 1: Figure S1).

These findings, especially the positivity for ALK protein, supported the diagnosis of IMT originating from the gastric wall. As well, metastatic tumor cells were found in 5/19 lymph nodes (Fig. 2f and g) and were positive for ALK on immunohistochemical staining (Fig. 2h and i). Fluorescent in situ hybridization confirmed the existence of ALK rearrangement in IMT tissues (Fig 2j). No *Helicobacter pylori* and EBV infection was found based on observation with oil immersion lens and chromogenic in situ hybridization of EBV-encoded small RNAs, respectively (Fig. 2k).

**Fig. 3:**
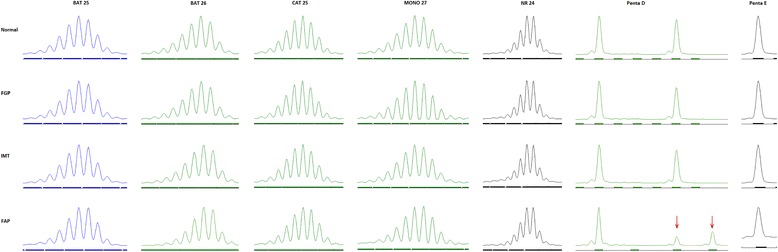
Microsatellite instability status of gastric fundic gland polyp (FGP), gastric inflammation myofibroblastic tumor (IMT) and familial adenomatous polyposis (FAP) tissue. Seven polymorphic markers, including BAT 25, BAT 26, CAT 25, MONO 27, NR 24, Penta D and Penta E, were used for microsatellite instability detection in the 3 tissue types as compared with normal colon tissue. Penta D in FAP was abnormal (*red arrow*)

**Fig. 4:**
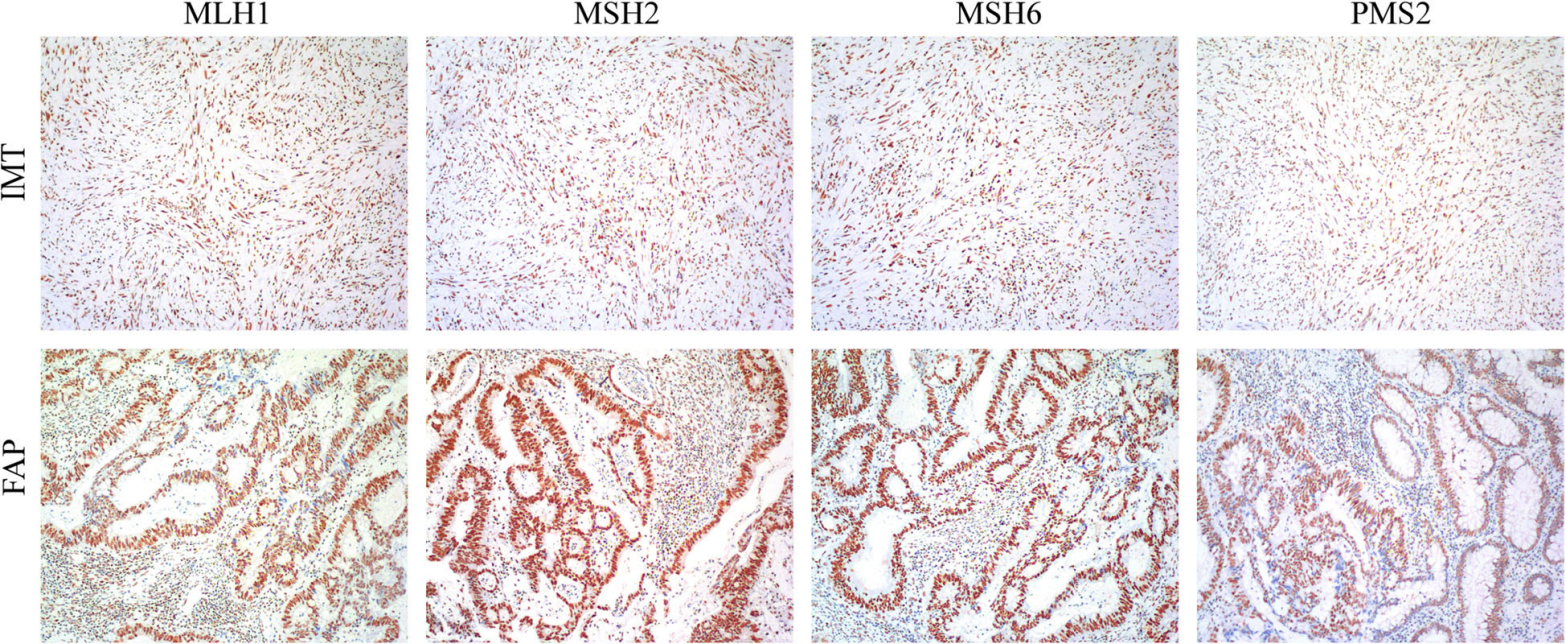
Mismatch repair system components normally expressed in gastric IMT and FAP. Components tested include MSH6, MSH2, MLH1 and PMS2

To explore the genetic relevance among IMT, FGPs and previous FAP tissue, we compared the ALK expression in the 3 tissue types and found IMT tissue positive but both FGP and FAP tissue negative for ALK protein. Then we investigated microsatellite instability status among the tissue types by testing 7 polymorphic markers, including BAT 25, BAT 26, CAT 25, MONO 27, NR 24, Penta D and Penta E, which are sensitive for microsatellite instability in colorectal cancer. Only Penta D expression was abnormal in FAP tissue (Fig. 3), which suggested that some atypical cells in FAP developed structure variation in the Penta D locus. On immunochemical analysis, IMT and FAP tissue was positive for mismatch repair-system components, including MSH6, MSH2, MLH1 and PMS2, which indicates their non-significant role in the development of IMT and FAP (Fig. 4).

## Discussion

IMT of the stomach, especially IMT with metastasis, in adults is extremely rare [3]. People with gastric IMT usually present non-specific symptoms and signs [9, 10]. Even with a thorough diagnostic examination, including CT, ultrasonography, and endoscopic biopsy, an accurate preoperative diagnosis is difficult. The current case showed typical spindle cells with prominent chronic inflammation background and was especially positive for ALK rearrangement but focally positive for CD34 and negative for CD117, S100, CD21 and CD23, which supports the clear pathological diagnosis of IMT and excludes all other possibilities.

The differential diagnosis of gastric IMT includes GIST, smooth muscle tumors, schwannoma, inflammatory fibroid polyps and inflammatory pseudotumor-like follicular dendritic cell sarcoma [11] and IgG4-related sclerosing disease. The typically inflammatory background of IMT is not shared with GIST. Immunohistochemically, GIST typically expresses CD117 but not ALK, whereas IMT is consistently negative for CD117. Gastric smooth-muscle neoplasm also does not have an inflammatory background and is diffusely positive for smooth muscle actin, desmin and caldesmon. Gastric schwannoma usually shows peripheral cuff-like lymphoid aggregation and expression of S-100 protein. Most inflammatory fibroid polyp cases are positive for CD34 and usually with increased eosinophils, and their stromal cells tend to condense around blood vessels to form whorled, perivascular cuffs, which are absent in IMT [12]. Lack of expression of CD21 and CD23 in IMT could exclude inflammatory pseudotumor-like follicular dendritic cell sarcoma [13, 14]. In addition, IgG4-related sclerosing disease could be ruled out because the IgG4/IgG ratio was <0.4 in our case (Additional file 1: Figure S1). So careful histology, immunohistochemistry, and clinical correlation are helpful for a correct diagnosis of gastric IMT.

Most FAP patients show gastric FGPs, which ranged from 12.5 to 88% of patients in a study published in 2008 [15], but FGPs with developing IMT have not been reported. IMT usually occurs after infection, trauma, surgical operation and inflammation, but the specific etiology remains unclear. EBVinfection [6] and immune dysregulation are associated with some cases of IMT [7]. Chromosomal variation is also hypothesized to contribute to IMT development. Approximately one-half of IMT cases show a cytogenetic translocation that activates *ALK*, thereby resulting in overexpression of ALK protein [8]. The fusion partners identified include *TPM3/4*, *CLTC*, and *RANBP2* [16]. A subset of ALK-negative IMT cases showed *ROS-1* and *PDGFRb* gene fusion [8]. Our case had no evidence of chronic gastric infection (such as *H. pylori* and EBV infection) and the IMT tissue was far from the previous surgical boundary, but ALK was positive in primary IMT tissue and metastatic lymph nodes but not FGPs and colon polyps, so translocation involving ALK at chromosome 2p23 may contribute to the development and progression of IMT but not the formation of FGP and FAP.

Because of the rarity of adult gastric IMT, especially in patients with a history of FAP, we tried to explore the genetic background among gastric IMT, gastric FGP and FAP. First, we performed microsatellite instability study of the 3 tissue types and found no shared abnormality. Second, we immunohistochemically tested the status of mismatch repair-system components, including MSH6, MSH2, MLH1 and PMS2, which were also normal. ALK was the only marker we found specifically expressed in IMT tissue and its metastatic lesion but not FAP and gastric FGP.

The biological behavior of IMT varies from benign to intermediate. Previously, ALK overexpression was found a favorable prognostic factor for IMT [17]. Coffin et al. found ALK reactivity associated with local recurrence but not distant metastasis [18]. Our ALK-positive case showed lymph node metastasis but not distant metastasis, which agrees with the Coffin et al. findings.

The recurrence rate is 25% for extrapulmonary IMT [19], so complete surgery is important for prognosis. Although the FGPs had no dysplasia signs in our case and FAP-associated FGPs rarely develop high-grade dysplasia and gastric adenocarcinoma [20], we still performed total gastric resection to avoid local IMT recurrence. Six-month follow-up showed no specific symptoms and long-term follow-up is ongoing.

## Conclusions

We describe the first case of ALK-positive gastric IMT with metastasis in an adult after total colectomy for FAP. Awareness of the inflammatory component and ALK rearrangement is important to distinguish IMT from other submucosal lesions. Immunohistochemically detecting ALK-positive tumor cells in lymph nodes is a reliable way to verify IMT metastasis. Further investigations are required to clarify possible a genetic basis for concurrence of IMT and FAP.

## Additional file

Additional file 1: Figure S1. Immunohistochemistry of protein expression of immunoglobulin G (IgG) and IgG4 in lymphocytes of gastric IMT. A: IgG, B: IgG4. (Immunohistochemical stain, ×100). (TIFF 1871 kb)

## Abbreviations

ALK: Anaplastic lymphoma kinase
CT: Computed tomography
EBV: Epstein-barr virus
FAP: Familial adenomatous polyposis
FDP: Fundic gland polyposis
GIST: Gastrointestinal stromal tumor
IgG: Immunoglobulin G
IgG4: Immunoglobulin G4
IMT: Inflammatory myofibroblastic tumor
SMA: Smooth muscle actin
