# Supplementary figures and images for: ALK-positive gastric inflammatory myofibroblastic tumor in an adult with familial adenomatous polyposis and diffuse fundic polyposis
Source: Diagn Pathol. 2017 Sep 18;12:68. doi: 10.1186/s13000-017-0645-0 (PMC5604288; doi:10.1186/s13000-017-0645-0)

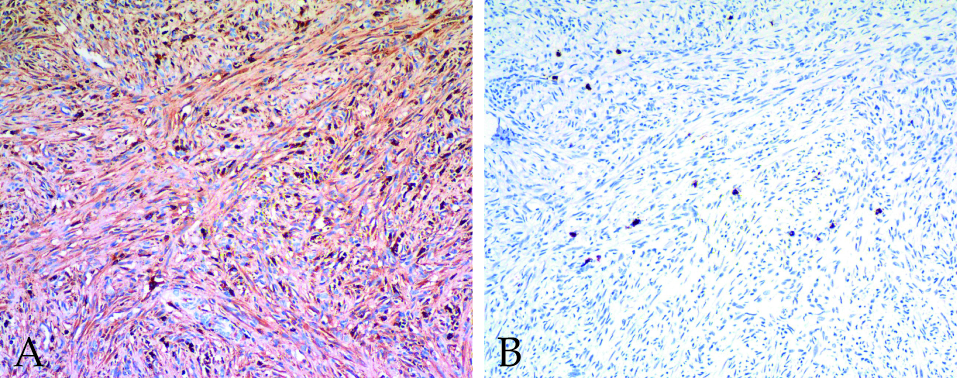

Supplement: Additional file 1: Figure S1. — Immunohistochemistry of protein expression of immunoglobulin G (IgG) and IgG4 in lymphocytes of gastric IMT. A: IgG, B: IgG4. (Immunohistochemical stain, ×100). (TIFF 1871 kb) [file 13000_2017_645_MOESM1_ESM.tif]
